# Highly Sensitive Zinc Oxide Nanorods for Non-Enzyme Electrochemical Detection of Ascorbic and Uric Acids

**DOI:** 10.3390/bios16030143

**Published:** 2026-03-01

**Authors:** Lesya V. Gritsenko, Zhaniya U. Paltusheva, Dinara T. Tastaibek, Khabibulla A. Abdullin, Zhanar K. Kalkozova, Maratbek T. Gabdullin, Juqin Zeng

**Affiliations:** 1 General Physics Department, Institute of Energy and Mechanical Engineering, Satbayev University, Satpayev Str., 22, Almaty 050013, Kazakhstan; 2 Institute of Applied Science & Information Technology, Nazarbayev Ave. 36-1, Almaty 050040, Kazakhstan; kh.abdullin@physics.kz (K.A.A.); zhanar.kalkozova@kaznu.edu.kz (Z.K.K.); 3 Department of Materials Science, Nanotechnology and Engineering Physics, Mining and Metallurgical Institute, Satbayev University, Satpayev Str., 22, Almaty 050013, Kazakhstan; dinaratastaibek@gmail.com; 4 National Nanotechnology Laboratory of Open Type (NNLOT), Al-Farabi Kazakh National University, Al-Farabi Ave., 71, Almaty 050040, Kazakhstan; 5 School of Materials Science and Green Technology, Kazakh-British Technical University, Tole Bi Street, 59, Almaty 050000, Kazakhstan; m.gabdullin@kbtu.kz; 6 Center for Sustainable Future Technologies, Istituto Italiano di Tecnologia, Via Livorno 60, 10144 Turin, Italy; juqin.zeng@polito.it; 7 Department of Applied Science and Technology, Politecnico di Torino, Corso Duca Degli Abruzzi 24, 10129 Turin, Italy

**Keywords:** enzyme-free electrochemical sensor, zinc oxide, ascorbic acid, uric acid, heat treatment, potential-controlled selectivity

## Abstract

In this study, an enzyme-free electrochemical sensor based on zinc oxide (ZnO) nanorods synthesized by the thermal decomposition of zinc acetate is presented. The suggested approach ensures simplicity, environmental friendliness, and scalability of the process without the use of an autoclave or high pressure. The morphology and structure of the samples are studied using SEM, TEM, XRD, Raman, FTIR, XPS, PL, and UV-Vis spectroscopy. It is found that heat treatment at 450 °C increases the degree of crystallinity, increases the size of crystallites, and reduces the concentration of surface defects, which leads to improved optical and electrochemical characteristics of the material. Beyond conventional sensitivity metrics, our study demonstrates that the selective detection of ascorbic acid (AA) and uric acid (UA) can be achieved by controlling the applied potential on a single ZnO electrode, an approach that leverages differences in redox energetics and surface interaction dynamics rather than complex surface functionalization. It is shown in this work that the synthesized ZnO samples subjected to heat treatment in air at 450 °C exhibit high sensitivity to ascorbic acid (9951.87 μA·mM^−1^·cm^−2^; LoD = 1.11 μM) at a potential of 0.2 V and to uric acid (5762.48 μA·mM^−1^·cm^−2^; LoD = 1.71 μM) in a phosphate buffer solution (pH 7) at a potential of 0.4 V with a linear range of 3 mM, offering a way to create simplified multicomponent electrochemical biosensors based on potential-controlled selectivity.

## 1. Introduction

Over the last few decades, the interdisciplinary fields of research on and synthesis of semiconductor nanostructured materials have been actively developed; their advantages are due to the materials’ unique physicochemical properties [1,2,3,4,5]. A^II^B^VI^-type semiconductors hold a prominent position [6,7,8,9,10,11,12]. A notable representative of this class is zinc oxide (ZnO). This compound is known for being electrically conductive, transparent, and exhibiting piezoelectric behavior. It possesses a wide band gap of 3.37 eV and an exciton binding energy of 60 meV. ZnO has many unique physical and chemical properties, such as high photostability, high chemical resistance, and a wide radiation absorption range. These attributes make it an essential functional material among metal oxides and explain its broad utilization in various fields, such as optical and electrochemical sensors, photocatalysts, photoresistors, light-emitting diodes, gas sensors, fluorescent materials, and solar cells [7,8,10,13,14,15,16,17,18]. Moreover, ZnO is notable for its environmentally friendly nature, simple synthesis, biocompatibility, non-toxic behavior, and biosafety, all of which contribute to its wide application in biological systems [6,7,15]. As a powder, it is frequently incorporated into products such as glass, cement, plastic, ceramics, and food as an additive [19,20].

There is a wide range of approaches for ZnO fabrication, including chemical vapor deposition (CVD) [21], pulsed laser deposition (PLD) [22], the hydrothermal route [23], the sol-gel technique [16,24], chemical bath deposition [8], green synthesis [25], and others. While the CVD and PLD methods are technically demanding and costly due to their reliance on specialized apparatus, wet-chemical approaches are generally simpler, more cost-effective, and capable of achieving high deposition rates without requiring complex instrumentation. To prepare a sample with high crystalline quality, the temperature and duration of synthesis play a major role, since very often high temperature entails an increase in the number of internal defects due to sintering between nanorods. The task of obtaining high-performance ZnO samples by environmentally friendly, simple, economical and controllable methods is urgent.

Currently, ZnO’s remarkable features have led to its extensive use in the biomedical field, particularly for detecting biomolecules, organic substances, acids, and similar com-pounds [7,12,13,16,26,27,28,29]. Examples include organic molecules such as ascorbic acid (vitamin C) and uric acid, which are vital for maintaining redox balance in cellular processes. AA is actively involved in strengthening the human immune system, neutralizing free radicals. It is characterized by antioxidant activity, which can prevent a number of diseases, such as neurodegenerative diseases, age-related muscle degeneration, mental illness, cataracts, cancer, hypertension, and others [30,31,32]. Ascorbic acid is especially important in fields such as cosmetology, pharmaceuticals, food quality control, and clinical diagnostics, where its detection and quantification are of great interest. UA acts as an antioxidant and oxygen-free-radical scavenger. Abnormal levels of UA are associated with various disorders, such as gout, diabetes, hypertension, Lesch–Nyhan syndrome, kidney and heart disease, and Parkinson’s and Alzheimer’s disease [33,34].

There are various methods for AA and UA detection, such as chemiluminescence, titrimetric analysis, UV spectrophotometry and liquid chromatography, colorimetry, and enzymatic assays. However, these approaches are expensive, complex, time-consuming, and involve tedious sample preparation procedures. Taking into account the importance and wide application of AA and UA, monitoring of AA and UA content should be considered as a necessary and relevant task in many areas of human endeavor. The main advantages of the electrochemical method are its high sensitivity, exceptional selectivity, high reproducibility and accuracy, low power consumption, ease of operation and cost-effectiveness, and the possibility of sensor miniaturization. While some electrochemical sensors can detect analytes only in alkaline media, those that demonstrate electrocatalytic activity and selectivity in neutral media are of particular interest. This is due to their physiological compatibility, enhanced analyte stability, and greater safety when analyzing biological and natural samples.

The development of highly sensitive, selective, and stable non-enzymatic electrochemical sensors for the detection of biologically significant compounds, such as ascorbic acid (AA) and uric acid (UA), remains an important task in materials science and bioanalytics. Among the various strategies for non-enzymatic electrochemical sensing, much of the literature focuses on material design to improve the sensitivity and limit of detection by incorporating conductive additives, graphene-base hybrid architectures [35,36,37], metal–organic frameworks [38], and transition metal oxides [39,40,41], as well as their composites with noble metals or biomass [42,43,44]. The sensitivity of such sensors varies widely, from moderate values of around 200–2000 μA·mM^−1^·cm^−2^ [36,38,42,43] to values exceeding 7000 μA·mM^−1^·cm^−2^ [26,35,37,40,44]. Many of these systems require complex multistep syntheses, the use of toxic reagents, high temperatures or pressures, and function primarily in acidic or alkaline environments, which limits their applicability in biological conditions. However, the intrinsic electrochemical selectivity achievable via the controlled polarization of a single sensing material remains comparatively underexplored, especially for simple metal oxide semiconductors such as ZnO. Traditional electrochemical sensor studies have often reported the simultaneous detection of multiple analytes through peak deconvolution or enhanced surface area without explicitly harnessing the applied potential as a tunable selectivity parameter [45,46,47].

This paper proposes a non-enzymatic sensor based on zinc oxide (ZnO) nanorods synthesized by the simple method of thermal decomposition of zinc acetate without the use of an autoclave or high pressure. This approach ensures environmental friendliness, the ability to control the particle size and morphology, scalability, and technological accessibility, which distinguishes it favorably from most previously described methods. Moreover, our work targets potential-controlled selectivity—the deliberate use of electrochemical potential windows to discriminate between redox-active biomolecules on the same ZnO electrode surface, under neutral conditions relevant to physiological samples. This approach shifts the sensing paradigm from materials-centric modifications to the electrochemical tuning of redox pathways, capitalizing on the differences in oxidation energetics, surface adsorption dynamics, and charge transfer properties. Unlike many systems that require an acidic or strongly alkaline environment for the effective oxidation of analytes [26,36], this sensor maintains high performance under physiologically relevant conditions. The significant increase in response indicates that the thermal treatment of zinc oxide in air leads to improved conductivity due to passivation of defects and the appearance of active centers that promote the oxidation process, which contribute to a noticeable improvement in the sensory properties of ZnO samples. Thus, this work not only advances the performance metrics of ZnO-based electrochemical sensors but also contributes to a novel mechanistic understanding of how applied potentials can impart selective electrocatalysis on oxide surfaces, which has important implications for the development of simple multi-analyte biosensor platforms.

## 2. Materials and Methods

### 2.1. Procedure

**Figure 1:**
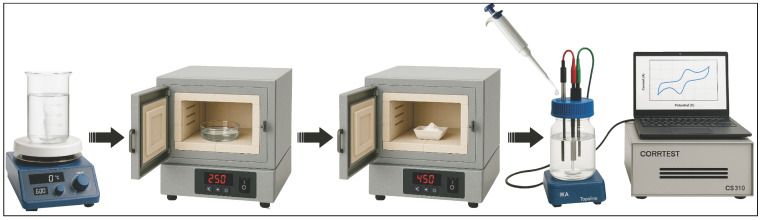
Scheme of synthesis and subsequent application of ZnO nanorods.

To obtain zinc oxide samples, the method of thermal decomposition of a zinc-containing ethanol solution was used. A total of 0.4 g of zinc acetate Zn(CH_3_COO)_2_ (Sigma Aldrich, 99.9%, St. Louis, MO, USA) dissolved in 10 mL of ethanol (C_2_H_5_OH, Sigma Aldrich, 99.8%, St. Louis, MO, USA) was used as the starting material. The solution was placed on a magnetic stirrer for one hour until the zinc acetate salts were completely dissolved. Synthesis of zinc oxide was carried out in a muffle furnace at 250 °C for 60 min, which resulted in the formation of ZnO sample in the form of fine powder (as grown). Some of the obtained zinc oxide samples were further heat-treated in air in a muffle furnace for one hour at 450 °C (after treatment). The thermal treatment was carried out in a programmable muffle furnace (Nabertherm GmbH, Lilienthal, Germany) under an ambient atmosphere. The annealing temperature was determined experimentally as the optimal temperature at which changes in the properties of synthesized ZnO samples became noticeable, with the prospect of their application as the basis for sensor devices. The general scheme for obtaining and using the synthesized ZnO samples is shown in Figure 1.

When zinc acetate is dissolved in ethanol, solvation and partial alcoholysis coordination occur without the formation of a complete chemical reaction. In an ethanolic medium, Zn^2+^ ions can undergo weak coordination with ethanol molecules. Upon thermal treatment at 250 °C in the presence of atmospheric oxygen, a sequence of physicochemical processes takes place, including the evaporation of ethanol followed by the thermal decomposition of zinc acetate. The decomposition reaction can be represented as

Zn(CH_3_COO)_2_ → ZnO + (CH_3_)_2_CO + CO_2_, (1)

where minor byproducts such as CH_3_COOH, CO, and CH_4_ may also be formed. Subsequently, the organic decomposition products are oxidized by atmospheric oxygen according to the reaction

(CH_3_)_2_CO + 4O_2_ → 3CO_2_ + 3H_2_O. (2)

As a result, the nanocrystalline ZnO remains in the beaker, while the organic components are completely removed through oxidative combustion. In this process, ethanol serves solely as a solvent and dispersing medium rather than as a reducing agent.

### 2.2. Characterization

The surface of zinc oxide nanorods was studied by scanning electron microscope (SEM, FEI Company, Quanta 200i 3D, FEI, Hillsboro, OR, USA) and transmission electron microscope (TEM, JEOL, JEM-1400 plus, Tokyo, Japan). A Fourier transform infrared absorption spectrophotometer (FTIR, Agilent Cary 630, Santa Clara, CA, USA) was used to detect various characteristic functional groups. The structural properties of the semiconductor sample were studied on an X-ray diffractometer (PANalitical X’pert MPD PRO, Almelo, The Netherlands) and by Raman spectroscopy (Solver Spectrum with a 473 nm solid-state exciting laser, NT-MDT, Zelenograd, Russia). The surface analysis of the material was characterized by XPS spectroscopy (NEXSA X-ray Photoelectron Spectrometer, Thermo Scientific, Waltham, MA, USA). In addition, the photoluminescence spectra (PL, Cary Eclipse spectrofluorimeter, Agilent, Santa Clara, CA, USA, under 300 nm excitation) and UV-vis optical density spectra (Agilent Technologies Cary Eclipse Fluorescence Spectrophotometer, Santa Clara, CA, USA) of the synthesized samples were studied. Electrochemical properties of ZnO samples were investigated using cyclic voltammetry (single-channel potentiostat–galvanostat, Corrtest CS310, Wuhan, China) and a three-electrode electrochemical cell containing an auxiliary platinum electrode and an Ag/AgCl reference electrode in addition to the working GCE (Corrtest CS310, Wuhan, China). All the measurements were carried out at room temperature. All the reagents were of analytical grade and used as received (Sigma-Aldrich, St. Louis, MO, USA). The electrochemical measurements were performed in 0.1 M phosphate buffer solution (PBS, pH 7.0).

## 3. Results and Discussion

### 3.1. Morphology and Structural and Optical Properties

**Figure 2:**
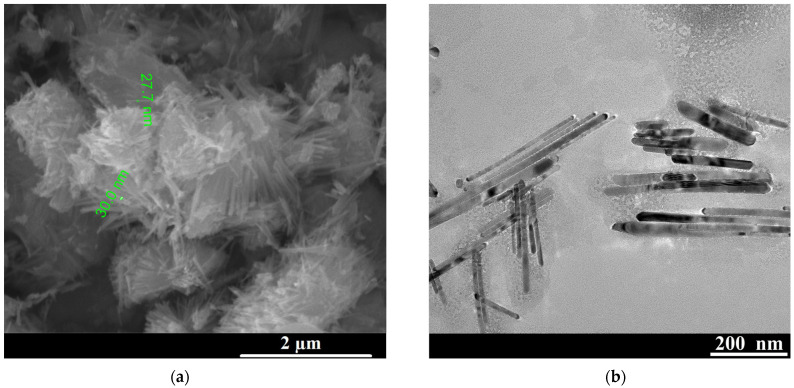
SEM (**a**) and TEM (**b**) images of the as-grown ZnO sample.

Investigation of the surface morphology of the initial ZnO samples by scanning electron microscopy (Figure 2a) and transmission electron microscopy (Figure 2b) showed that the synthesized samples consist of a dense cluster of one-dimensional, chaotically oriented nanorods of elongated shape, with an average length varying from 200 to 500 nm and a diameter of 20–40 nm. The rod clusters are characterized by a rather porous structure, indicating a high specific surface area, which is important for sensing and catalytic applications. The transmission electron microscopy (Figure 2b) confirmed that the individual nanorods have the shape of one-dimensional rods with lengths of several hundred nanometers and diameters of several tens of nanometers. After thermal annealing at 450 °C, no significant visual change was found in the SEM or TEM images, possibly due to the wide variation in the values of geometric parameters caused by differences in the local growth kinetics of the nanorods. The annealing temperature of 450 °C was chosen as an optimal compromise between effective defect passivation, removal of residual organic species, and preservation of the nanorod morphology. Lower temperatures are insufficient to significantly improve crystallinity and electronic transport, while higher temperatures are known to induce sintering and, consequently, a decrease in the surface area of ZnO nanostructures [48,49].

**Figure 3:**
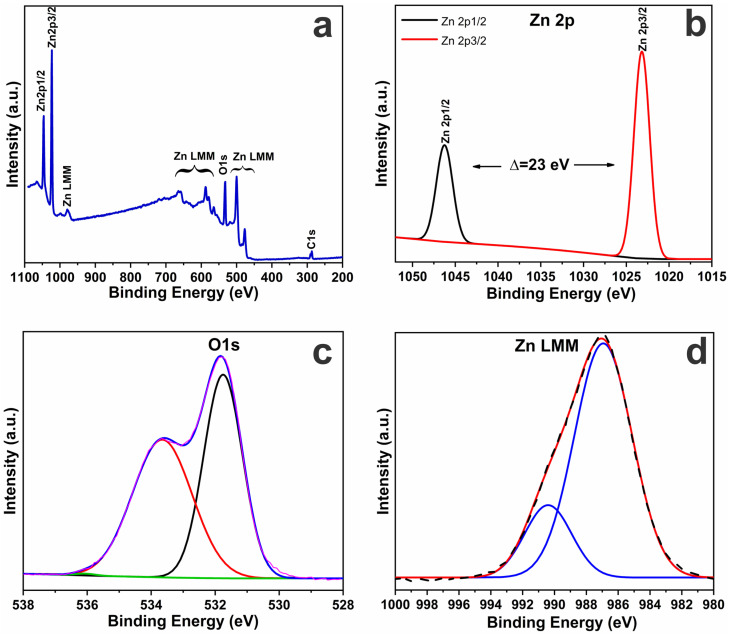
XPS spectra of ZnO samples: Overall XPS survey spectra (**a**), high-resolution XPS spectra of Zn 2p (**b**), O 1s (**c**) and Zn LMM Auger spectra (**d**).

Analysis of the XPS spectrum of the initial ZnO sample allowed for identification of a number of characteristic peaks corresponding to the main elements included in the composition of the studied material (Figure 3a). The characteristic peaks of Zn 2p, registered at 1023.08 eV and 1046.08 eV, belong to the Zn^2+^ 2p_3_/_2_ and Zn^2+^ 2p_1_/_2_ states, respectively, which indicates spin–orbit splitting of the Zn 2p level and confirms the presence of divalent zinc (Figure 3b). The peaks are symmetric and not shifted, indicating the purity of the ZnO phase. The difference between these peaks is approximately 23 eV, indicating the spin–orbit splitting characteristic of zinc ions in oxidation degree +2. The obtained values are in good agreement with the literature data for zinc oxide (ZnO) [50,51,52]. The presence of a well-defined double cleavage and the energy position of the peaks indicate that zinc is present mainly in the form of Zn^2+^, without a significant contribution of metallic zinc or other compounds. This indicates the high quality of synthesis and the purity of the ZnO phase [50,52,53]. Additionally, Auger peaks of Zn LMM were observed in the (665.08–564.08) eV and (500–477.08) eV regions, which also confirms the presence of zinc in the composition of the investigated material. The Zn 2p and Zn LMM Auger spectra confirm the exclusive presence of Zn^2+^ characteristic of ZnO. No evidence of metallic Zn was detected. Therefore, the electrochemical processes discussed in this work are governed by surface-mediated electron transfer rather than Zn/ZnO phase conversion.

The XPS spectrum of the O 1s region for the investigated ZnO sample was decomposed into three main components, reflecting the different chemical states of oxygen in the material structure (Figure 3c). The O 1s peak is represented by two components located at 531.78 eV and 533.68 eV, which correspond, respectively, to lattice oxygen (O^2−^) and surface hydroxyl groups (–OH), as well as adsorbed forms of oxygen (O^−^, O^2−^). The main peak with a maximum at ~531.75 eV (black line) is due to oxygen bound to the metal oxide crystal lattice (O^2−^ in ZnO positions), which confirms the presence of an ordered Zn–O lattice. The second component at ~533.65 eV (red line) indicates the presence of surface defects, such as hydroxyl groups, water molecules, and oxygen vacancies, which are characteristic of nanostructured ZnO. The third component at ~536.06 eV (green line) indicates the minor presence of adsorbed water on the surface. The presence of hydroxyl groups and water is typical for sol-derived materials. The total approximating profile (blue line) agrees well with the experimental data (lilac line), which confirms the correctness of the performed spectrum decomposition. The pronounced high-energy shoulder indicates a significant contribution of surface and defect states of oxygen, which may be related to the high specific surface area of the material [50,51].

The presence of C 1s peaks is due to the residual organic compounds used in the synthesis steps (e.g., from ethanol or zinc acetate). The spectrum in the Zn LMM region shown in Figure 3d corresponds to the Auger transition characteristic of zinc in the observed range of 986–988 eV. This transition is due to the L_3_M_4,5_M_4,5_ process during which an electron from the L (2p) level fills a vacancy formed by photoelectron emission accompanied by an Auger electron emission involving the M (3s, 3p) levels.

Auger peaks are sensitive to the chemical environment and valence state of an element, so the spectral shape and energy of a given peak can be used as an independent tool to confirm the composition of a material. Characterization of the shape and position of the peak indicates the presence of zinc exclusively in the Zn^2+^ state characteristic of zinc oxide (ZnO). Metal zinc (Zn^0^), unlike ZnO, is characterized by a shift of the Auger peak to a lower energy region and a different shape of the curve.

In this case, the peak energy value (~987 eV) coincides with the literature data published for pure ZnO [50,51], which also confirms the absence of metallic zinc phases in the sample. The obtained approximating curve (red line) shows good agreement with the original experimental data (black line), which confirms the correctness of the processing and high reliability of the interpretation of the results. Thus, the use of Auger analysis in combination with the main peaks of Zn 2p can improve the accuracy of zinc valence state identification and serves as an important complement to a traditional binding level analysis.

**Figure 4:**
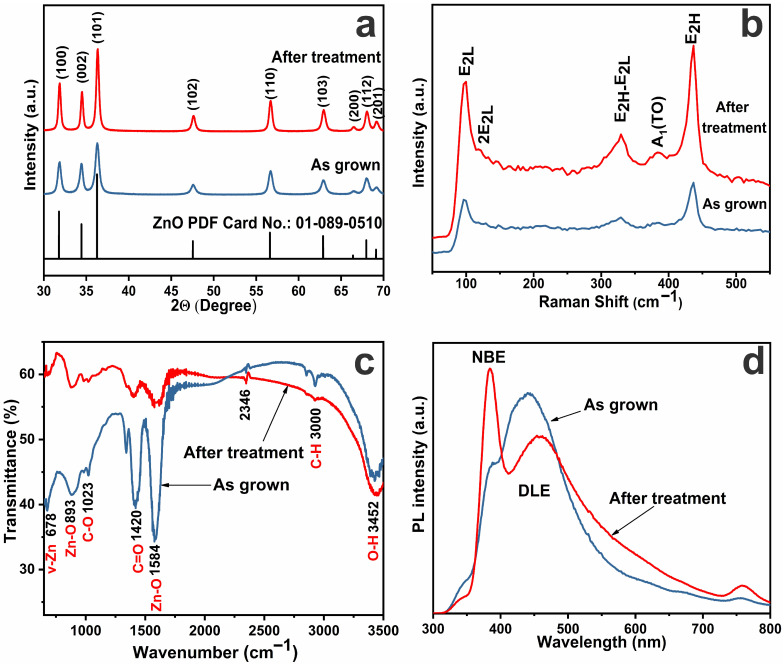
Structural and optical properties of ZnO samples: XRD spectra (**a**), Raman spectra (**b**), FTIR spectra (**c**), PL spectra (**d**).

The results of XRD analysis of both the synthesized ZnO samples and those subjected to subsequent annealing in air at 450° C are shown in Figure 4a. All the peaks in the diffractograms correspond to the hexagonal structure of wurtzite ZnO (JCPDS map No. 89-0510), with lattice parameters: a = 3.25 Å, b = 3.25 Å, c = 5.21 Å [54,55,56]. The coincidence of the peaks in the spectra of the grown samples with the reference spectrum indicates their phase purity. It is noted that in the original sample the peaks are less intense and broader; some of them are weakly expressed or blurred, which may indicate the small size of crystallites, the presence of internal defects, and lower crystallinity. After thermal annealing, the peaks become more intense (especially along the directions (100), (002), and (101)) and narrower, which indicates crystallite enlargement, a reduction in defects and internal stresses, and an increase in the degree of crystallinity. Strengthening of weakly expressed peaks indicates a more complete development of a crystalline structure.

Thus, the XRD analysis confirms that heat treatment at 450 °C leads to a significant improvement in the crystalline structure: it promotes increased crystallinity, reorganization of atoms into a more stable structure, elimination of amorphous and defective regions, and improved orientation. Estimation of crystallite sizes along the (101) direction uses Scherrer’s formula [55]:

D = K⋅λ/β⋅cosθ,(3)

where D is the average crystallite size (in nanometers), K = 0.9 is the shape constant, λ = 0.15406 nm is the wavelength of Cu Kα radiation, β is the peak width at half height (FWHM), and θ is the Bragg angle. This shows that for the initial ZnO sample, the rod diameter is approximately 25 nm; after heat treatment it is ~42 nm, which is consistent with the SEM and TEM results.

Figure 4b shows the Raman spectra of the investigated zinc oxide samples. Zinc oxide possesses a wurtzite structure (hexagonal P63mc), which is characterized by the following first-order optically active modes: E_2_(low) around ~100 cm^−1^, which characterizes Zn-sublattice vibrations; E_2_(high) around ~437 cm^−1^, which characterizes the O-sublattice vibrations; and A_1_(TO) around ~380 cm^−1^ is the transverse optical mode, as well as combined modes and superharmonics, such as 2E_2L_, E_2H_-E_2L_, etc. [57,58,59]. In the spectra of the original sample all modes are present, but their intensities are relatively low. The modes E_2_(low) (~100 cm^−1^) and E_2_(high) (~437 cm^−1^) are weakly expressed. The presence of broad peaks indicates low crystallinity and presence of defects and internal stresses in the crystal. In addition, an elevated background level is observable, which may be evidence of an amorphous phase or interphase boundaries. It is noticeable that after thermal treatment the intensity of E_2L_ and E_2H_ peaks increased significantly, a more distinct A_1_(TO) peak appeared, and additional peaks became more pronounced—2E_2L_ and E_2H_-E_2L_ [57]—which indicates the presence of two- and three-phonon processes that intensified after annealing. All the characteristic peaks became narrower, indicating an increase in the crystallinity of the material. The decrease in the background signal also indicates a decrease in the defect level and an improvement in the crystalline structure. Thus, the Raman spectroscopy confirms the XRD results: annealing at 450 °C significantly improves the crystalline quality of the grown ZnO samples.

To investigate the effect of heat treatment on the optical properties of zinc oxide, the optical absorption spectra were studied (Figure S1a) and the dependence of (αhν)^2^ on the photon energy hν was plotted according to the Tauc method [60] to estimate the band gap width (Figure S1b). The figures show that the original and annealed samples exhibited a sharp increase in the absorption coefficient in the UV region (300–400 nm), which corresponds to the fundamental absorption edge of ZnO and indicates the direct-band nature of the transition. It was noted that the absorption in the UV region of the annealed sample (red line) increased, indicating an increase in crystallinity and a decrease in defect density. Moreover, after annealing at 450 °C, the absorption edge shifted slightly towards shorter wavelengths (Figure S1a), indicating an increase in the band gap. This was also accompanied by a slight decrease in the absorption intensity in the visible region (400–700 nm), which can be attributed to a decrease in the density of defect states in the band gap region, such as oxygen vacancies or interstitial zinc atoms responsible for sub-band levels.

The points of intersection of the tangents drawn to the linear parts of the curves with the energy axis (hν, eV) in Figure S1b allowed us to determine by the Tauc method the band gap values of the studied samples. For the original sample, the value of Eg was 3.27 eV, while for the sample heat-treated at 450 °C the value increased to 3.33 eV; the error was ~0.02 eV. This increase can be attributed to the decrease in the concentration of defect states and the improvement in the crystal structure of the material after annealing.

FTIR analysis of the as-grown ZnO samples was performed to detect different functional groups. Figure 4c shows the FTIR transmission spectra of the original (blue line) and annealed at 450 °C (red line) samples, showing the presence of peaks characteristic of different functional groups of ZnO in the range of 500–3500 cm^−1^. It can be observed that after heat treatment the intensity of most of the peaks decreased significantly and some peaks disappeared completely. The increase in the transmittance of the red line indicates a decrease in absorption, i.e., a decrease in the concentration of certain functional groups. The 3200–3700 cm^−1^ broad band is associated with vibrations of –OH^−^ groups (hydroxyl groups, water, and surface hydroxyls) [61,62].

The original sample shows an intense, broad peak at ~3400 cm^−1^, indicating the presence of a significant amount of water or hydroxyl groups. After treatment at 450 °C, the intensity of this peak is markedly reduced, indicating the removal of water and/or dihydroxylation of the ZnO surface. In the region ~1400–1600 cm^−1^, peaks corresponding to C=O, C-H, C-C vibrations, ionized carbonate and organic residues are often observed [61,62,63]. The initial sample has pronounced peaks at 1420 cm^−1^ and 1560 cm^−1^, which significantly weaken or disappear after annealing, indicating the decomposition of organic contaminants or precursor residues on the surface. The 1000–1300 cm^−1^ region corresponds to C-O, C-N, S=O and other organic residues. The decrease in peak intensity in this region also indicates the removal of organic impurities after heat treatment. In the region less than 1000 cm^−1^, there are characteristic bands of Zn-O vibrations (~500–600 cm^−1^). They are present in both spectra, but in the spectrum of the original sample they are more pronounced. After thermal treatment these peaks may slightly shift or decrease due to reorganization of the structure or ordering of the crystal lattice. Residual Zn-O vibrations are retained, confirming that the base material (ZnO) is not destroyed. Thus, heat treatment at 450 °C leads to the removal of hydroxyl and organic groups, such as moisture, precursors, and surface contaminants, contributing to the purification of the ZnO surface, which is particularly important for sensing and optoelectronic applications. This suggests the structural and chemical ordering of the material, confirming the results of XRD and Raman analyses.

The photoluminescence (PL) spectra of the synthesized zinc oxide samples, presented in Figure 4d, show the characteristic peaks responsible for intrinsic (NBE) and impurity (DLE) photoluminescence [64,65]. It can be seen that the spectrum of the original sample shows a broad peak in the region of 450–650 nm due to the presence of surface and bulk defects, such as oxygen vacancies (VOs), zinc vacancies (VZns), interstitial atoms and defect complexes. After thermal treatment, the intensity of impurity in the PL spectra decreases markedly, while the NBE peak in the UV region (~380 nm) associated with the radiative recombination of free excitons is enhanced. These changes indicate a decrease in the density of defect states and an improvement in the crystallinity of the material after annealing [16,66].

Beyond improving the optical quality, the structural and electronic modifications induced by annealing at 450 °C have direct consequences for the electrocatalytic behavior of ZnO nanorods. The enhanced crystallinity evidenced by the XRD and Raman spectroscopy reduces intergranular barriers and facilitates long-range electron transport, while the suppression of deep-level defect states observed in the PL spectra minimizes charge trapping and recombination losses. Simultaneously, the FTIR analysis confirms the removal of insulating organic residues and excess hydroxyl species, leading to a chemically cleaner and electronically more accessible ZnO surface. These combined effects establish a favorable balance between surface reactivity and electronic conductivity, which is essential for efficient electron transfer during the electrochemical oxidation of ascorbic and uric acids.

### 3.2. Electrochemical Characterization

The electrochemical measurements were carried out using a three-electrode cell in 0.1 M PBS (pH 7.0) at room temperature. A cleaned glassy carbon electrode (GCE 2 mm in diameter) was used as the working electrode. To improve the contact between the synthesized ZnO samples and the working GCE, the flat end surface of the GCE with the ZnO sample was pressed tightly against a substrate of porous ceramic plate. The ZnO nanorod powder was not pelletized or dispersed in a polymer matrix. Instead, a binder-free contact configuration was employed, in which the powder was mechanically pressed onto a polished glassy carbon electrode using a porous ceramic support. This configuration was selected to evaluate the intrinsic electrocatalytic activity of the ZnO nanorods without interference from binders or solvents. Platinum and Ag/AgCl electrodes were taken as the counter electrode and reference electrode, respectively. The concentrations of AA and UA were varied from 0 mM to 3 mM.

**Figure 5:**
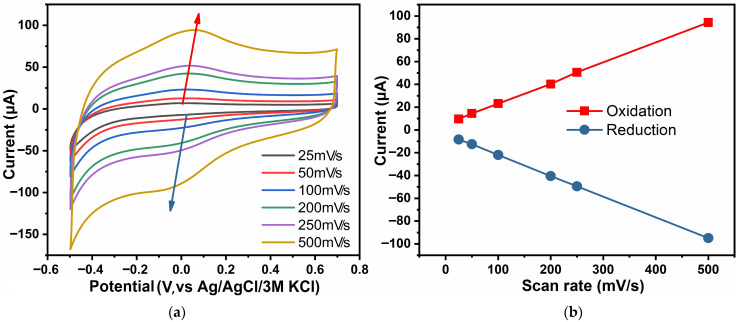
CV of ZnO samples in 0.1 M PBS at different scan rates (**a**) and plot of anodic and cathodic peak currents as function of scan rates (**b**).

Figure 5 shows the CV of ZnO samples in 0.1 M PBS at different scan rates—25, 50, 100, 200, 250 and 500 mV/s from the inner graph to the outer (Figure 5a)—as well as a plot of anodic and cathodic peak currents as a function of scan rate (Figure 5b). Cyclic voltammograms were recorded in the potential range −0.5 to 0.7 V vs. Ag/AgCl.

The study was carried out to analyze the electrokinetics of oxidation and reduction processes on the electrode surface at different scan rates. It was noted that both the anodic (oxidation) and cathodic (reduction) peak currents increased with an increasing scan rate; at the same time, the peaks of anodic peaks were slightly shifted to the region with higher potentials, while the peaks of cathodic peaks were shifted to lower potentials. The general shape of the curve was generally preserved at all rates, indicating the stability of the electrochemical system. The linear dependence of the maxima of anodic and cathodic currents on the scan rate (Figure 5b) indicates an adsorption-controlled electrochemical process [67,68]. Consequently, the ZnO-based sensor is stable over a wide range of scan rates, which makes it promising for sensing applications.

**Figure 6:**
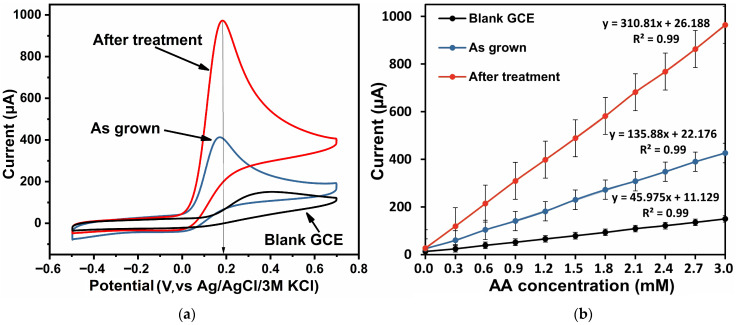
Electrochemical characteristics: CV of ZnO samples and blank GCE in 0.1 M PBS in the presence of 3 mM AA (**a**); plot of anodic and cathodic peak currents as a function of AA concentration (**b**).

**Figure 7:**
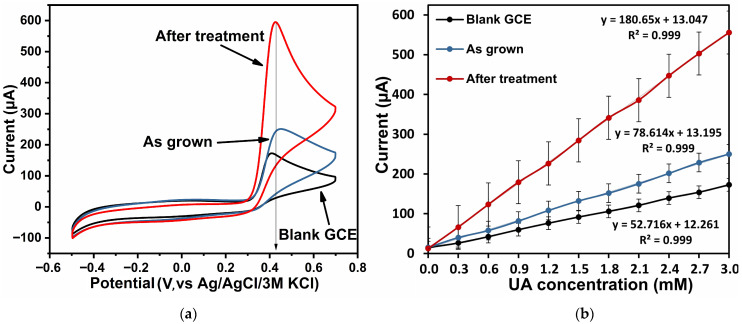
Electrochemical characteristics: CV of ZnO samples and blank GCE in 0.1 M PBS in the presence of 3 mM UA (**a**); plot of anodic and cathodic peak currents as a function of UA concentration (**b**).

The electrochemical properties of the synthesized samples were studied with respect to the detection of ascorbic acid (Sigma Aldrich, 98%) and uric acid (Sigma Aldrich, 99%), the concentration of each of which was varied in the range of (0–3) mM for correct comparison, which corresponds to the clinical range of real samples. A schematic redox mechanism of AA and UA on ZnO nanorods is shown in Figure S2. Figure 6a and Figure 7a show CV plots for the as-grown ZnO, the ZnO after treatment, and the blank GCE in the presence of 3 mM AA and UA in 0.1 M PBS at a scan rate of 100 mV/s, respectively.

The maximum oxidation currents were recorded at a potential of 0.2 V for AA detection and at 0.4 V for UA detection. The lower oxidation potential of AA (~0.2 V) compared to UA (~0.4 V) can be explained by differences in the molecular redox energetics and surface interactions with ZnO. Ascorbic acid forms strong hydrogen bonds with surface hydroxyl groups and directly interacts with Zn^2+^ surface sites, facilitating rapid electron transfer into ZnO conduction bands. In contrast, uric acid exhibits slower charge transfer kinetics and requires higher anodic polarization due to its more complex molecular structure and higher intrinsic oxidation potential. Figure 6b and Figure 7b show the calibration dependences of oxidation currents of the considered ZnO samples and blank GCE on AA and UA concentrations in 0.1 M PBS, reflecting their sensitivity towards the selected analytes.

The enhanced oxidation currents observed for both AA and UA on the ZnO electrodes annealed at 450 °C can thus be directly correlated with the structural and optical evolution presented in Figure 4. In particular, defect passivation and improved crystallinity lower the charge transfer resistance and enable the faster extraction of electrons from the adsorbed analyte molecules. While AA benefits additionally from strong surface interactions and hydrogen bonding with ZnO, UA oxidation, being kinetically more demanding, relies predominantly on improved electronic conductivity and reduced surface contamination. This explains why annealing leads to a more pronounced relative improvement in UA sensitivity, despite its higher oxidation potential. To support the choice of the 450 °C annealing temperature as optimal for establishing the role of thermal treatment in modulating ZnO defect chemistry and electrochemical characteristics, Figure S3a shows a comparison of the electrochemical characteristics of ZnO samples—as grown and annealed in air at 350 °C, 450 °C, and 550 °C in 0.1 M PBS in the presence of 3 mM AA—as well as a plot of anodic and cathodic peak currents as a function of AA concentration for these samples (Figure S3b).

The analysis of cyclic voltammetry shows that the blank GCE had a weak response to the presence of analytes. The as-grown zinc oxide samples showed sensitivities of 4257.35 µA·mM^−1^ cm^−2^ and 2496.27 µA·mM^−1^·cm^−2^ towards AA and UA, respectively. After treatment, the ZnO samples had the highest sensitivity of the series considered: 9951.87 µA·mM^−1^·cm^−2^ (LoD 1.11 µM) and 5762.48 µA·mM^−1^·cm^−2^ (LoD 1.71 µM) with respect to AA and UA, respectively. The LoD was calculated using the ratio

LoD = 3.3 *σ*/*S*,

where *σ* is the standard deviation of the baseline (*n* = 10) and *S* is the slope of the calibration curve.

The significant enhancement in the response indicates that heat treatment of zinc oxide in air resulted in passivation of defects and appearance of active centers promoting the oxidation process, which contributed to the marked improvement in the sensing properties of ZnO samples. Their excellent linearity, high sensitivity and a wide range of linear responses make the proposed ZnO samples the most promising and competitive for use as the basis of electrochemical sensors.

**Figure 8:**
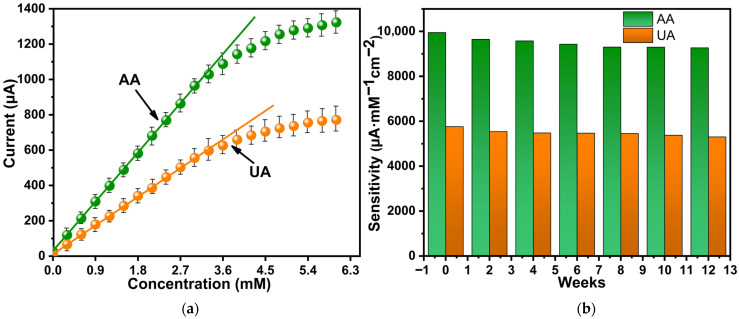
Response of after-treatment ZnO-based sensor to the presence of AA and UA in 0.1 M PBS: linear response region (**a**), stability evaluation over time (**b**).

In order to determine the linear response region of the presented ZnO-based electrochemical sensor for ascorbic and uric acid detection, measurements were carried out when the concentration of acids in the phosphate-buffered solution was varied from 0 to 6 mM. As shown in Figure 8a, using the after-treatment ZnO samples for both acids (AA is the green line, UA is the orange line), the linear region was on the order of 3 mM. The prepared after-treatment ZnO samples showed stable AA and UA detection signals with less than 5% deviation compared to the original value after 12 weeks of storage at room temperature (Figure 8b). This means that after treatment the ZnO samples generated a chemical signal with excellent stability.

**Figure 9:**
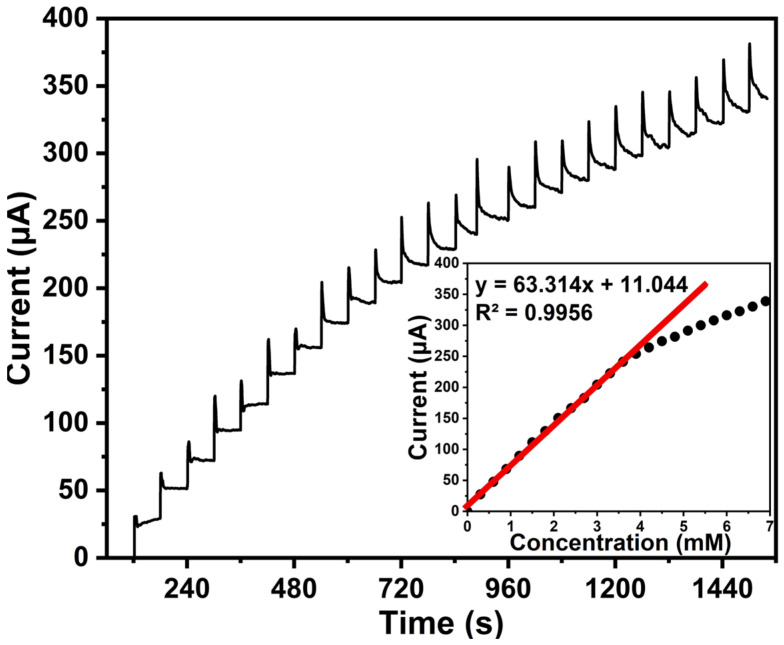
Amperometric response of after-treatment ZnO-based sensor in 0.1 M PBS at a potential of 0.2 V.

To evaluate the sensitivity and response dynamics of after-treatment ZnO samples, an amperometric assay was performed to track the response of successive AA additions in 0.1 M PBS at a potential of 0.2 V, as shown in Figure 9. Throughout the experiment 60 µM of ascorbic acid was added sequentially to the solution.

With each addition of AA, the current spiked and then stabilized, showing a step-like response pattern. This demonstrates the rapid response of the sensor to analyte addition and its ability to reach a steady-state current within a short time after each addition. The sensor based on the after-treatment ZnO samples showed a high response rate to the presence of AA in solution. The response time was less than 3 s. The linearity between the peak oxidation current of AA and its concentration is shown in the inset of Figure 9. The linear response range of after-treatment ZnO samples with an increasing AA concentration was ~3 mM.

To better understand the redox processes on the ZnO samples–GCE surface, the electrochemical impedance spectroscopy (EIS) was measured in the frequency range of 0.1 ÷ 10^5^ Hz at a bias voltage of 0.2 V when AA was added and at 0.4 V when UA was added in 0.1 M PBS. Figure S4 shows the Nyquist plots for the original (Figure S4a) and for the ZnO sample heat-treated at 450° C in air (Figure S4b). The insets show the Randle circuit, where R1 is the equivalent series resistance of the electrolyte solution, R2 is the charge transfer resistance, and CPE1 is the constant phase element modeling the capacitive behavior. The inserted tables show the measurement values of the circuit elements.

For the as-grown ZnO sample in the presence of 3 mM AA, the EIS curve has the shape of a semicircle, typical for processes limited by charge transfer, with parameters: R1 = 5.00 Ohm·cm^2^ (error 0.67%); R2 = 989.9 Ohm·cm^2^ (error 1.68%); and CPE1 = 0.86 (error 0.37%) (Figure S4a). The high value of R2 indicates a hindered charge transfer process at the interface.

The heat-treated ZnO in the presence of 3 mM AA also shows a semicircular curve, but with a smaller amplitude, indicating improved electrode properties (Figure S4b), with parameters R1 = 4.25 Ohm·cm^2^ (error 0.65%), which is slightly lower than that of the as-grown sample, possibly due to the better conductivity in the solution after heat treatment; and CPE1 = 0.91 (error 0.36%) and R2 = 575.07 Ohm·cm^2^ (error 1.29%), which are significantly lower than those of as-grown ZnO, indicating an improvement in charge transfer. The Nyquist plots for the ZnO-based electrodes measured at 0.4 V in the presence of 3 mM uric acid in 0.1 M buffer solution are shown in Figure S4c,d. The EIS curve of as-grown ZnO sample has a pronounced semi-elliptical character with a large radius typical of limited charge transfer, with R1 = 4.86 Ohm·cm^2^ (error 1.06%), CPE1 = 0.87 (error 0.46%), and R2 = 5252.5 Ohm·cm^2^ (error 5.14%). The shape of the EIS curve for the heat-treated ZnO at 450 °C is also semi-elliptical, but with a lower height, indicating improved conductive properties in the solution.

The charge transfer resistance, R2 = 4070.9 Ohm·cm^2^ (error 4.48%), decreased compared to the value of the original sample by ~22.5%, indicating an improvement in the charge transfer kinetics. The behavior of CPE and R1 remains almost unchanged, implying the stability of the interface structure and electrolyte. The charge transfer resistance R2 of modified GCE with the heat-treated ZnO in the presence of AA is lower than in the presence of UA, indicating better oxidation of ascorbic acid compared to uric acid.

Thus, during the thermal treatment of ZnO at 450 °C, the rods are enlarged and recrystallized, the number of structural defects and dislocations is reduced, and stable, active crystalline planes are formed on the surface, favorable for electrochemical reactions and electron mobility and the charge transfer efficiency is increased. The ZnO surface becomes cleaner, allowing it to adsorb analytes more easily. Ascorbic acid forms hydrogen bonds with the surface OH- groups and can interact with the Zn^2+^ on the crystal surface. Under moderate annealing, a balance between sufficient surface defects and improved conductivity is maintained. More efficient adsorption increases the local AA concentration, resulting in a higher current. The surface and intergranular charge traps (dangling bonds, broken bonds, and oxygen groups) are eliminated or passivated by the air oxygen. This reduces the height of the intergranular barriers; hence, electrons can tunnel or diffuse more easily between the nanorods and the charge transfer resistance (R2) is reduced. The charge transfer resistance affects the ability of the electrode to transfer electrons efficiently, which in turn determines the current strength at a given analyte concentration. The lower this resistance, the higher the electrochemical activity and sensitivity of the sensor. As a consequence, electrons are transferred away from the analyte faster, increasing the current and enhancing the sensitivity of the sensor to the analyte. In addition, the clean, crystallized ZnO surface is less susceptible to fluctuations and can be reused repeatedly without signal degradation if the material is immobilized on the electrode surface.

For creating an enzyme-free sensor, it is especially important to study its selectivity towards interfering compounds that occur simultaneously in physiological fluids, such as blood or human serum. The selectivity of the ZnO nanorod-based electrode toward ascorbic acid (AA) and uric acid (UA) was evaluated by amperometric measurements in 0.1 M phosphate buffer solution (pH 7.0) in the presence of potentially coexisting species. The investigated interferents were selected to represent common low-molecular-weight compounds frequently encountered in biological and physiological environments, including carbohydrates (glucose, maltose), organic acids (lactic acid), nitrogen-containing metabolites (urea), and inorganic salts (NaCl).

**Figure 10:**
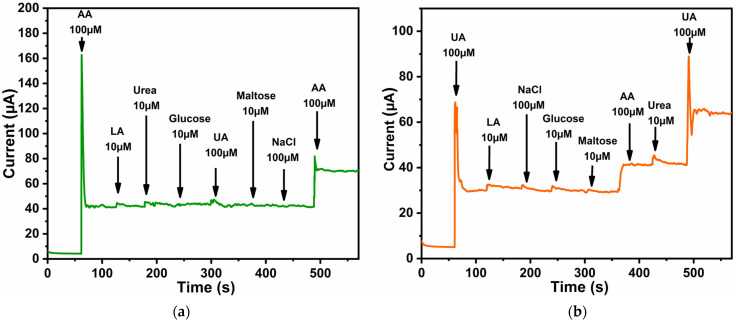
Amperometric response of modified ZnO samples in 0.1 M PBS to the successive addition of AA (100 μM), UA (100 μM), urea (10 μM), NaCl (100 μM), lactic acid (10 μM), glucose (10 μM), and maltose (10 μM): (**a**) measurements performed at 0.2 V vs. Ag/AgCl, corresponding to the preferential oxidation of AA; (**b**) measurements performed at 0.4 V vs. Ag/AgCl, corresponding to the dominant oxidation of UA.

The amperometric responses were recorded at fixed potentials of 0.2 V and 0.4 V versus Ag/AgCl, corresponding to the characteristic oxidation potentials of AA and UA, respectively (Figure 10). At 0.2 V, a pronounced and reproducible current response was observed upon the addition of AA, whereas the subsequent introduction of glucose, maltose, lactic acid, urea, and NaCl resulted in negligible changes in the current. This behavior indicates that, under these conditions, the ZnO-based electrode exhibits preferential sensitivity toward AA oxidation (Figure 10a).

At 0.4 V, the electrode displayed a dominant response to UA, while a weaker but non-negligible response to AA was also detected (Figure 10b). This partial overlap reflects the intrinsically similar redox potentials of AA and UA on metal oxide surfaces and highlights the necessity of potential control and calibration when simultaneous detection is required. Thus, selectivity is achieved through controlled polarization windows rather than through inherent molecular exclusivity. Importantly, none of the other tested species produced a significant electrochemical signal at this potential, suggesting minimal interference from electrochemically inactive or weakly active compounds within the investigated concentration range. It should be emphasized that the present selectivity assessment focuses on the electrochemical interference from small, low-molecular-weight species.

The observed selectivity arises from a combination of potential discrimination and surface-specific interactions between the ZnO and target analytes, which is due to their different surface affinity. AA forms hydrogen bonds with the –OH groups on the ZnO surface, while UA interacts less strongly and requires a higher activation energy. Interferent concentrations were chosen to reflect or exceed their upper physiological limits in serum, providing a conservative estimate of selectivity. Kinetic suppression of interfering substances is related to the fact that neutral molecules (glucose, urea) do not exhibit favorable adsorption or redox kinetics on ZnO at these potentials, thus enabling selective detection by controlled-potential tuning. Electrochemically inactive molecules, such as alcohols, amino acids, and macromolecular species (e.g., proteins), were not investigated in this study. Such species primarily influence sensor performance through surface fouling or matrix effects rather than direct faradaic processes, and their impact requires dedicated experiments involving real biological or complex samples. Overall, the results demonstrate that the ZnO nanorod-based electrode enables potential-selective discrimination between AA and UA in neutral aqueous media. While complete electrochemical orthogonality between these analytes is not expected due to their comparable oxidation behavior, the observed response patterns confirm that controlled-potential operation and appropriate calibration strategies allow for reliable detection within mixed-analyte environments. These findings support the suitability of thermally treated ZnO nanorods as a robust materials platform for enzyme-free electrochemical sensing, with further validation in complex matrices forming the focus of future studies.

## 4. Conclusions

This work demonstrates that zinc oxide nanorods synthesized via a simple and environmentally benign thermal decomposition route can serve as an effective platform for the potential-controlled, enzyme-free electrochemical sensing of biologically relevant analytes. Beyond achieving high sensitivity, the study reveals that electrochemical selectivity can be actively governed by the applied potential, rather than relying solely on complex material modification or biochemical recognition elements. Thermal treatment at 450 °C plays a decisive role in establishing this behavior by simultaneously improving crystallinity, stabilizing the ZnO surface, reducing defect-mediated charge trapping, and enhancing the charge transfer kinetics at the semiconductor–electrolyte interface. These structural and electronic modifications translate directly into a more predictable and reproducible electrochemical response, enabling selective oxidation of ascorbic acid at lower potentials and uric acid at higher potentials on the same electrode surface. Importantly, the results highlight that potential-controlled selectivity represents a powerful yet underutilized design principle for enzyme-free biosensors, particularly for metal oxide semiconductors. By exploiting the intrinsic differences in the redox energetics and surface interactions of target molecules, selective detection can be achieved without sacrificing simplicity, stability, or scalability. This approach is especially advantageous for biosensing in neutral aqueous environments, where enzymatic systems often suffer from a limited operational lifetime and chemical instability. The demonstrated strategy opens new perspectives for the development of multi-analyte electrochemical sensors based on a single sensing material, where analyte discrimination is achieved through controlled electrochemical polarization rather than material complexity. Such a paradigm is highly attractive for low-cost, miniaturized, and wearable sensing technologies aimed at biomedical diagnostics, food quality monitoring, and environmental analysis.

## Data Availability

Data is contained within the article or Supplementary Materials.

## Supplementary Materials

The following supporting information can be downloaded at: https://www.mdpi.com/article/10.3390/bios16030143/s1, Figure S1: Optical density spectra (a), Tauc plot (b); Figure S2: A schematic redox mechanism of AA (a) and UA (b) on ZnO nanorods; Figure S3: Electrochemical characteristics: CV of ZnO samples (as grown and annealed in air at 350 °C, 450 °C, and 550 °C) and blank GCE in 0.1 M PBS in the presence of 3 mM AA (a), plot of anodic and cathodic peak currents as a function of AA concentration (b); Figure S4: EIS of ZnO samples in the frequency range 0.1 ÷ 10^5^ Hz: ZnO as grown, 3 mM AA (a); ZnO after treatment, 3 mM AA (b); ZnO as grown, 3 mM UA (c); ZnO after treatment, 3 mM UA (d).

## Abbreviations

The following abbreviations are used in this manuscript:

AA	Ascorbic Acid
UA	Uric Acid
GCE	Glassy Carbon Electrode

## References

[1] Mirzaei A., Ansari H.R., Shahbaz M., Kim J.-Y., Kim H.W., Kim S.S. (2022). Metal Oxide Semiconductor Nanostructure Gas Sensors with Different Morphologies. Chemosensors.

[2] Kawakami R., Miyaji Y., Yanagiya S., Shirai A., Koinkar P., Furube A., Nakano Y., Niibe M. (2025). Enhanced photocatalytic activity of TiO_2_/Au/TiO_2_/Au stacked nanostructures synthesized via sputtering and subsequent annealing. Appl. Surf. Sci..

[3] Markhabayeva A.A., Kalkozova Z.K., Nemkayeva R., Yerlanuly Y., Anarova A.S., Tulegenova M.A., Tulegenova A.T., Abdullin K.A. (2024). Construction of a ZnO heterogeneous structure using Co_3_O_4_ as a co-catalyst to enhance photoelectrochemical performance. Materials.

[4] Serik A., Idrissov N., Baratov A., Dikov A., Kislitsin S., Daulbayev C., Kuspanov Z. (2024). Recent Progress in Photocatalytic Applications of Electrospun Nanofibers: A Review. Molecules.

[5] Mussabek G., Zhylkybayeva N., Baktygerey S., Yermukhamed D., Taurbayev Y., Sadykov G., Zaderko A.N., Lisnyak V.V. (2023). Preparation and characterization of hybrid nanopowder based on nanosilicon decorated with carbon nanostructures. Appl. Nanosci..

[6] Maafa I.M. (2025). Potential of Zinc Oxide Nanostructures in Biosensor Application. Biosensors.

[7] Zhang L. (2020). Electrochemical Detection of Ascorbic Acid in Citrus Juices using Mn-doped ZnO nanorods modified graphene oxide. Int. J. Electrochem. Sci..

[8] Kedruk Y.Y., Contestabile A., Zeng J., Fontana M., Laurenti M., Gritsenko L.V., Cicero G., Pirri C.F., Abdullin K.A. (2023). Morphology Effects on Electro- and Photo-Catalytic Properties of Zinc Oxide Nanostructures. Nanomaterials.

[9] Permiakov N., Maraeva E., Bobkov A., Mbwahnche R., Moshnikov V. (2023). Investigation of the Conductive Properties of ZnO Thin Films Using Liquid Probes and Creation of a Setup Using Liquid Probes EGaIn for Studing the Conductive Properties of Thin Films. Technologies.

[10] Bakranova D., Seitov B., Bakranov N. (2022). Preparation and Photocatalytic/Photoelectrochemical Investigation of 2D ZnO/CdS Nanocomposites. ChemEngineering.

[11] Yang C., Zhang H., Zhang H. (2025). Preparation and mechanism study of highly sensitive NH3 gas sensor based on Au-modified CdS nanorods. Appl. Surf. Sci..

[12] Akrami Z.E., Sohouli E. (2025). Preparation of an ultra-sensitive electrochemical sensor for morphine measurement using the ZnS/Mil-125 nanocomposite. J. Alloys Compd..

[13] Sha R., Basak A., Maity P.C., Badhulika S. (2022). ZnO nano-structured based devices for chemical and optical sensing applications. Sens. Actuators Rep..

[14] Witkowski B.S., Pietruszka R., Gieraltowska S., Wachnicki L., Przybylinska H., Godlewski M. (2017). Photoresistor based on ZnO nanorods grown on a p-type silicon substrate. Opto-Electron. Rev..

[15] Mohamed K.M., Benitto J.J., Vijaya J.J., Bououdina M. (2023). Recent Advances in ZnO-Based Nanostructures for the Photocatalytic Degradation of Hazardous, Non-Biodegradable Medicines. Crystals.

[16] Tolubayeva D.B., Gritsenko L.V., Kedruk Y.Y., Aitzhanov M.B., Nemkayeva R.R., Abdullin K.A. (2023). Effect of hydrogen plasma treatment on the sensitivity of ZnO based electrochemical non-enzymatic biosensor. Biosensors.

[17] Khan M.W.A., Shaalan N.M., Ahmed F., Sherwani S., Aljaafari A., Alsukaibi A.K.D., Alenezi K.M., Al-Motair K. (2024). Gas Sensing Performance of Zinc Oxide Nanorods Fabricated via Ochradenus baccatus Leaf. Chemosensors.

[18] Fallahazad P., Eshraghi M.J. (2025). Effect of reduced graphene oxide on the performance of ZnO thin film/pyramidal silicon heterostructure solar cells. Mater. Lett..

[19] Sheikh M., Pazirofteh M., Dehghani M., Asghari M., Rezakazemi M., Valderrama C., Cortina J.-L. (2020). Application of ZnO nanostructures in ceramic and polymeric membranes for water and wastewater technologies: A review. Chem. Eng. J..

[20] Zhou X.-Q., Hayat Z., Zhang D.-D., Li M.-Y., Hu S., Wu Q., Cao Y.-F., Yuan Y. (2023). Zinc Oxide Nanorods: Synthesis, Characterization, Modification, and Applications in Food and Agriculture. Processes.

[21] Phan T.-L., Cuong L.V., Lam V.D., Dang N.T. (2024). Various CVD-grown ZnO nanostructures for nanodevices and interdisciplinary applications. Beilstein J. Nanotechnol..

[22] Khlifi N., Ihzaz N., Toulemonde O., Dandre A., Labrugère-Sarroste C., Bessadok M.N., Lemine O.M., El Mir L. (2024). Cobalt-doped ZnO nanorods and PLD-deposited thin film forms: Structure, optical properties and nature of magnetic anisotropy. RSC Adv..

[23] Majid F., Bashir M., Bibi I., Raza A., Ezzine S., Alwadai N., Iqbal M. (2022). ZnO nanofibers fabrication by hydrothermal route and effect of reaction time on dielectric, structural and optical properties. J. Mater. Res. Technol..

[24] Abdullin K.A., Bakranov N.B., Ismailov D.V., Kalkozova J.K., Kumekov S.E., Podrezova L.V., Cicero G. (2014). Composite materials based on nanostructured zinc oxide. Semiconductors.

[25] Benzarti Z., Neiva J., Faia P., Silva E., Carvalho S., Devesa S. (2025). Novel Green Synthesis Route of ZnO Nanoparticles for Dielectric Applications. Nanomaterials.

[26] Nithya M. (2015). Electrochemical Sensing of Ascorbic Acid on ZnO-decorated Reduced Graphene Oxide Electrode. J. Biosens. Bioelectron..

[27] Abamecha A., Yimer A.A., Muleta G.G., Kitte S.A. (2025). Electrochemical Determination of Ascorbic Acid at Ag_2_S-CuO-ZnO Ternary Nanocomposite Modified Glassy Carbon Electrode. Sci. Afr..

[28] John J.F., Dhinasekaran D., Subashchandran S. (2024). ZnO/NiFe_2_O_4_ heterostructure on nickel foam for the electrochemical detection of uric acid. Mater. Chem. Phys..

[29] Masrat S., Nagal V., Khan M., Ahmad A., Alshammari M.B., Alam S., Nakate U.T., Lee B., Mishra P., Bhat K.S. (2023). Electrochemical Sensing of Uric Acid with Zinc Oxide Nanorods Decorated with Copper Oxide Nanoseeds. ACS Appl. Nano Mater..

[30] Guo D., Liao Y., Na J., Wu L., Yin Y., Mi Z., Fang S., Liu X., Huang Y. (2024). The Involvement of Ascorbic Acid in Cancer Treatment. Molecules.

[31] Maekawa T., MiyakeMasaji T., Uemoto T. (2022). Diverse antitumor effects of ascorbic acid on cancer cells and the tumor microenvironment. Front. Oncol..

[32] Lim J.C., Arredondo M.C., Braakhuis A.J., Donaldson P.J. (2020). Vitamin C and the Lens: New Insights into Delaying the Onset of Cataract. Nutrients.

[33] Gherghina M.-E., Peride I., Tiglis M., Neagu T.P., Niculae A., Checherita I.A. (2022). Uric Acid and Oxidative Stress—Relationship with Cardiovascular, Metabolic, and Renal Impairment. Int. J. Mol. Sci..

[34] Xu L., Li C., Wan T., Sun X., Lin X., Yan D., Li J., Wei P. (2025). Targeting uric acid: A promising intervention against oxidative stress and neuroinflammation in neurodegenerative diseases. Cell Commun. Signal.

[35] Banavath R., Abhinav A., Srivastava R., Bhargava P. (2022). Highly sensitive ascorbic acid sensors from EDTA chelation derived nickel hexacyanoferrate/graphene nanocomposites. Electrochim. Acta.

[36] Khattak N.S., Ara L., Shah L.A., Ullah R., Rehman T.U. (2024). Fabrication of non-enzymatic and highly sensitive electrochemical ascorbic acid sensor based on GO/Ag/PMMA nanocomposites. Inorg. Chem. Commun..

[37] Zhao Y., Qin J., Xu H., Gao S., Jiang T., Zhang S., Jin J. (2019). Gold nanorods decorated with graphene oxide and multi-walled carbon nanotubes for trace level voltammetric determination of ascorbic acid. Microchim. Acta.

[38] Wei C., Wang Z., Hu Y., Huang J., Zhang Y., Wang H., Liu Q., Yu Z. (2024). Layer-by-layer growth of Cu_3_(HHTP)_2_ films on Cu(OH)_2_ nanowire arrays for high performance ascorbic acid sensing. Biosens. Bioelectron..

[39] Rahman M.M., Rana M.S., Minami H., Rahman M.M., Rahman M.A., Alam M.A., Ahmad H. (2025). The efficiency of an aminated nanocrystalline cellulose stabilized binary Ag–ZnO nanocomposite as an electrode platform for electrochemical sensing of ascorbic acid. Mat. Adv..

[40] Alam M.M., Balkhoyor H.B., Asiri A.M., Karim M.R., Chani M.T., Rahman M.M. (2020). Fabrication of ascorbic sensor acid with Co_3_O_4_·Fe_2_O_3_ nanosphere materials by electrochemical technique. Surf. Interfaces.

[41] Masrat S., Nagal V., Khan M., Moid I., Alam S., Bhat K.S., Khosla A., Ahmad R. (2022). Electrochemical Ultrasensitive Sensing of Uric Acid on Non-Enzymatic Porous Cobalt Oxide Nanosheets-Based Sensor. Biosensors.

[42] Yin Y., Zhao J., Qin L., Yang Y., He L. (2016). Synthesis of an ordered nanoporous Fe_2_O_3_/Au film for application in ascorbic acid detection. RSC Adv..

[43] Ahmed J., Faisal M., Algethami J.S., Alsaiari M., Jalalah M., Harraz F.A. (2024). CeO·ZnO@biomass-derived carbon nanocomposite-based electrochemical sensor for efficient detection of ascorbic acid. Anal. Biochem..

[44] Karim M.R., Jayed M., Laskar M.Z., Bhuyan M.M., Islam M.S., Hayami S., Rahman M.M. (2023). Enhancement of functional surface and molecular dynamics at Pt-rGO by spacer 1,6-hexanediamine for precise detection of biomolecules: Uric acid as a specimen. Sens. Diagn.

[45] Pan Y., Zuo J., Hou Z., Huang Y., Huang C. (2020). Preparation of Electrochemical Sensor Based on Zinc Oxide Nanoparticles for Simultaneous Determination of AA, DA, and UA. Front. Chem..

[46] Fernandes D.M., Costa M., Pereira C., Bachiller-Baeza B., Rodríguez-Ramos I., Guerrero-Ruiz A., Freire C. (2014). Novel electrochemical sensor based on N-doped carbon nanotubes and Fe_3_O_4_ nanoparticles: Simultaneous voltammetric determination of ascorbic acid, dopamine and uric acid. J. Colloid Interface Sci..

[47] Somashekar M.N., Dhanalakshmi M., Nagamani T.S., Subhas Chandra T., Sharanakumar T.M., Ravikumar C.R. (2025). Photocatalytic and electrochemical sensor detection of ascorbic and uric acid using novel plant extract green synthesis of CaO nanoparticles. Sens. Int..

[48] Choi J.W., Lee C.M., Park C.H., Lim J.H., Park G.C., Joo J. (2019). Effect of Annealing Temperature on Morphology and Electrical Property of Hydrothermally-Grown ZnO Nanorods/p-Si Heterojunction Diodes. J Nanosci Nanotechnol..

[49] Yermakov M., Pshenychnyi R., Opanasyuk A., Gnatenko Y., Bukivskij P., Bukivskii A., Klymov O., Muñoz-Sanjosé V., Gamernyk R. (2025). The effect of annealing on the structural, optical, electrical and photoelectric properties of ZnO/NiO heterostructures. Appl. Surf. Sci. Adv..

[50] Rauwel E., Galeckas A., Rauwel P., Sunding M.F., Fjellvåg H. (2011). Precursor-Dependent Blue-Green Photoluminescence Emission of ZnO Nanorods. J. Phys. Chem..

[51] Zuo J., Erbe A. (2010). Optical and electronic properties of native zinc oxide films on polycrystalline Zn. Phys. Chem. Chem. Phys..

[52] Morales C., del Campo A., Méndez J., Prieto P., Soriano L. (2020). Re-Oxidation of ZnO Clusters Grown on HOPG. Coatings.

[53] Henderson J.D., Payne B.P., McIntyre N.S., Biesinger M.C. (2025). Enhancing Oxygen Spectra Interpretation by Calculating Oxygen Linked to Adventitious Carbon. Surf. Interface Anal..

[54] Das A., Deka T., Kumar P.M., Bhagavathiachari M., Nair R.G. (2022). Ag-modified ZnO nanorods and its dual application in visible light-driven photoelectrochemical water oxidation and photocatalytic dye degradation: A correlation between optical and electrochemical properties. Adv. Powder Technol..

[55] Gurgur E., Oluyamo S.S., Adetuyi A.O., Omotunde O.I., Okoronkwo A.E. (2020). Green synthesis of zinc oxide nanorods and zinc oxide–silver, zinc oxide-copper nanocomposites using Bridelia ferruginea as biotemplate. SN Appl. Sci..

[56] Guo W., Chen K., Zhang H. (2024). Synthesis and characterization of ZnO hexagonal sheets wrapped MoS_2_ sphere for tri-ethylamine gas sensing application. Ceram. Int..

[57] Song Y., Zhang S., Zhang C., Yang Y., Lv K. (2019). Raman Spectra and Microstructure of Zinc Oxide irradiated with Swift Heavy Ion. Crystals.

[58] Rajalakshmi M., Arora A.K., Bendre B.S., Mahamuni S. (2000). Optical phonon confinement in zinc oxide nanorods. J. Appl. Phys..

[59] Aljaafari A., Ahmed F., Awada C., Shaalan N.M. (2020). Flower-like ZnO Nanorods Synthesized by Microwave-Assisted One-Pot Method for Detecting Reducing Gases: Structural Properties and Sensing Reversibility. Front. Chem..

[60] Makuła P., Pacia M., Macyk W. (2018). How To Correctly Determine the Band Gap Energy of Modified Semiconductor Photocatalysts Based on UV–Vis Spectra. J. Phys. Chem. Lett..

[61] da Silva-Neto M.L., de Oliveira M.C.A., Dominguez C.T., Lins R.E.M., Rakov N., de Araújo C.B., de Souza Menezes L., de Oliveira H.P., Gomes A.S.L. (2019). UV random laser emission from flexible ZnO-Ag-enriched electrospun cellulose acetate fiber matrix. Sci. Rep..

[62] Vivek C., Balraj B., Thangavel S. (2019). Structural, optical and electrical behavior of ZnO@Ag core–shell nanocomposite synthesized via novel plasmon-green mediated approach. J. Mater. Sci. Mater. Electron..

[63] Mahalakshmi S., Hema N., Vijaya P.P. (2020). In Vitro Biocompatibility and Antimicrobial activities of Zinc Oxide Nanorods (ZnO NPs) Prepared by Chemical and Green Synthetic Route- A Comparative Study. BioNanoScience.

[64] Kumar S., Pandey J., Tripathi R., Chauhan R. (2023). Photoluminescence Investigations and Band Gap Engineering in Environment Friendly ZnO Nanorods: Enhanced Water Treatment Application and Defect Model. ACS Omega.

[65] Galdámez-Martinez A., Santana G., Güell F., Martínez-Alanis P.R., Dutt A. (2020). Photoluminescence of ZnO Nanowires: A Review. Nanomaterials.

[66] Abdullin A.K., Cicero G., Gritsenko L.V., Kumekov S.E., Markhabaeva A.A. (2017). Effect of annealing and hydrogen plasma treatment on the luminescence and persistent photoconductivity of polycrystalline ZnO films. J. Appl. Phys..

[67] Ma G., Shi Q., Hou X., Peng Y., Liu Q. (2024). An electrochemical sensor for simultaneous voltammetric detection of ascorbic acid and dopamine enabled by higher electrocatalytic activity of co-modified MCM-41 mesoporous molecular sieve. Front. Sustain. Food Syst..

[68] Han E., Pan Y.Y., Li L., Cai J.R. (2023). Bisphenol a detection based on nano gold-doped molecular imprinting electrochemical sensor with enhanced sensitivity. Food Chem..

