# Supplementary material for: Highly Sensitive Zinc Oxide Nanorods for Non-Enzyme Electrochemical Detection of Ascorbic and Uric Acids
Source: Biosensors (Basel). 2026 Mar 1;16(3):143. doi: 10.3390/bios16030143 (PMC13024462; doi:10.3390/bios16030143)
Supplement: Supplementary file 1 [file biosensors-16-00143-s001.zip › biosensors-4143350-supplementary.pdf]

biosensors

Supplementary Materials

Highly Sensitive Zinc Oxide Nanorods for Non-Enzyme Electrochemical Detection of Ascorbic and Uric Acids

Lesya V. Gritsenko [1,2,*], Zhaniya U. Paltusheva [3,*], Dinara T. Tastaibek [3], Khabibulla A. Abdullin [2,4], Zhanar K. Kalkozova [2,4], Maratbek T. Gabdullin [5] and Juqin Zeng [6,7]

1 General Physics Department, Institute of Energy and Mechanical Engineering, Satbayev University, Satpayev Str., 22, Almaty 050013, Kazakhstan
2 Institute of Applied Science & Information Technology, Nazarbayev Ave. 36-1, Almaty 050040, Kazakhstan; kh.abdullin@physics.kz (K.A.A.); zhanar.kalkozova@kaznu.edu.kz (Z.K.K.)
3 Department of Materials Science, Nanotechnology and Engineering Physics, Mining and Metallurgical Institute, Satbayev University, Satpayev Str., 22, Almaty 050013, Kazakhstan; dinaratastaibek@gmail.com
4 National Nanotechnology Laboratory of Open Type (NNLOT), Al-Farabi Kazakh National University, Al-Farabi Ave., 71, Almaty 050040, Kazakhstan
5 School of Materials Science and Green Technology, Kazakh-British Technical University, Tole Bi Street, 59, Almaty 050000, Kazakhstan; m.gabdullin@kbtu.kz
6 Center for Sustainable Future Technologies, Istituto Italiano di Tecnologia, Via Livorno 60, 10144 Turin, Italy; juqin.zeng@polito.it
7 Department of Applied Science and Technology, Politecnico di Torino, Corso Duca Degli Abruzzi 24, 10129 Turin, Italy
* Correspondence: l.gritsenko@satbayev.university (L.V.G.); zhaniya.paltusheva@gmail.com (Z.U.P.)

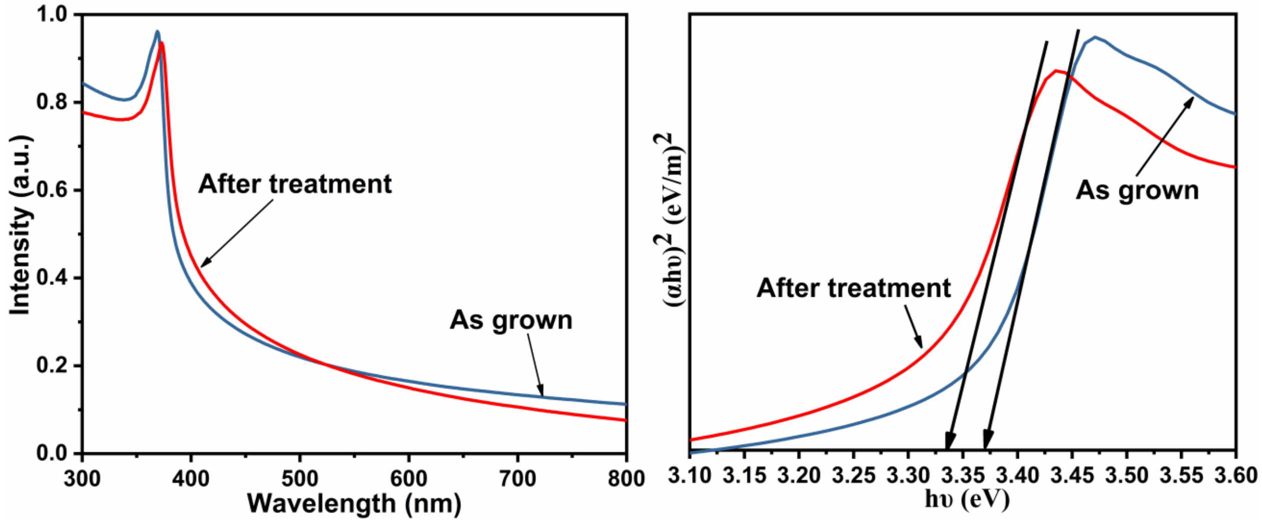

(**a**) (**b**)

Figure S1. Optical density spectra (**a**), Tauc plot (**b**).

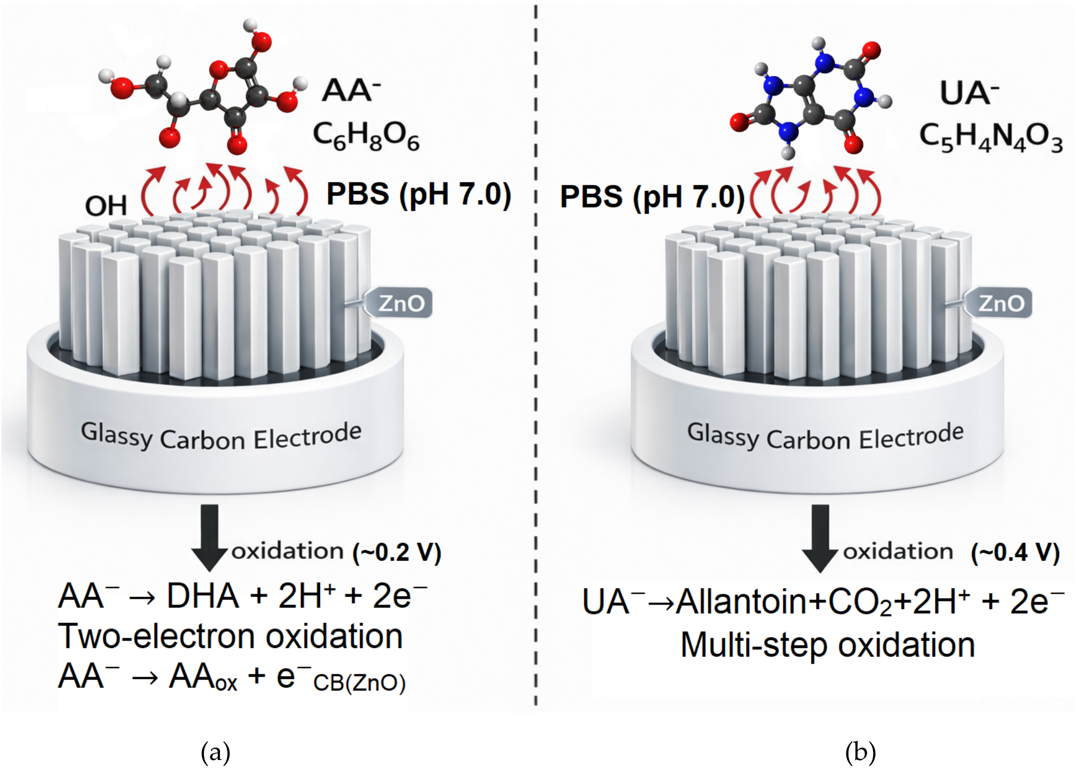

Figure S2. A schematic redox mechanism of AA (**a**) and UA (**b**) on ZnO nanorods.

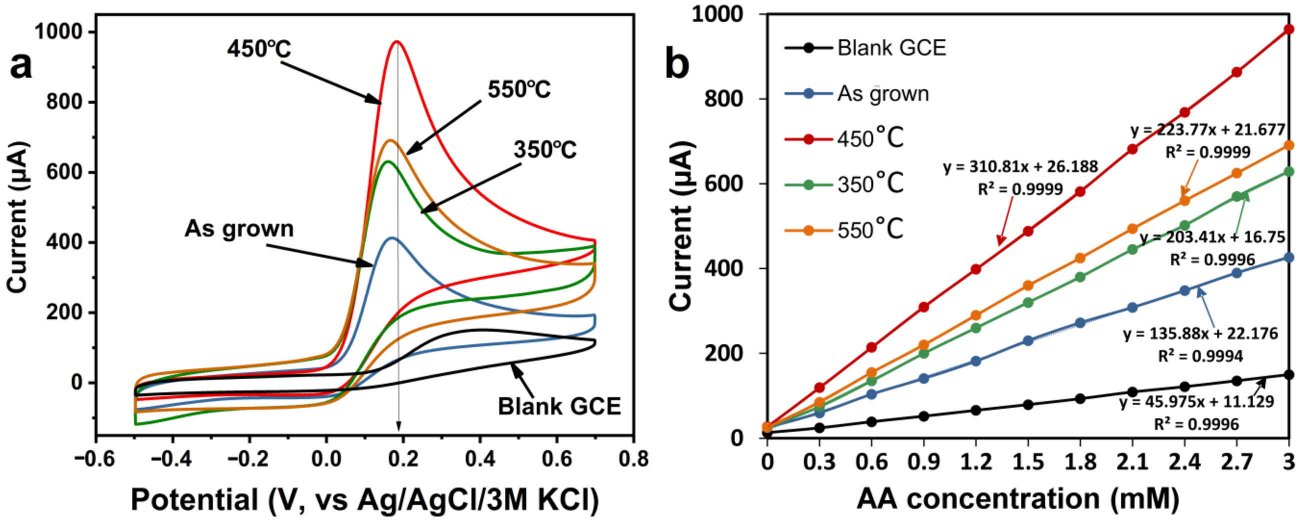

Figure S3. Electrochemical characteristics: CV of ZnO samples (as grown, annealed in air at 350 °C, 450 °C, and 550 °C) and blank GCE in 0.1 M PBS in the presence of 3 mM AA (**a**), plot of anodic and cathodic peak currents as a function of AA concentration (**b**).

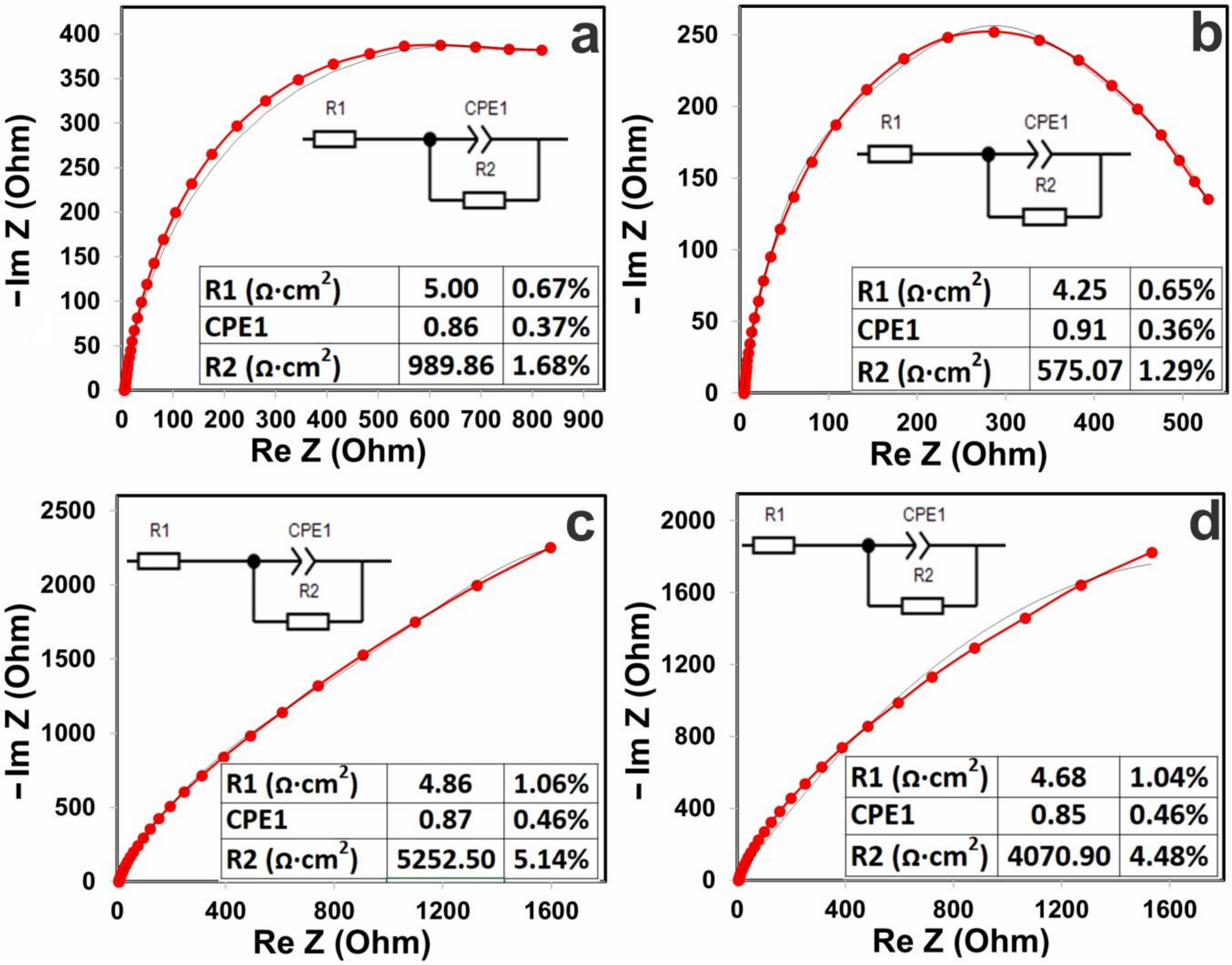

Figure S4. EIS of ZnO samples in the frequency range 0.1÷10^5 Hz: ZnO as grown, 3mM AA (**a**); ZnO after treatment, 3mM AA (**b**); ZnO as grown, 3mM UA (**c**); ZnO after treatment, 3mM UA (**d**).
